# Superior Replacement of Medicinal Gel With Ayurvedic Nanogel as a Coupling Medium for Electrotherapeutic Treatment of Osteoarthritis: A Review Article

**DOI:** 10.7759/cureus.28658

**Published:** 2022-09-01

**Authors:** Medhavi V Joshi, Pratik Phansopkar

**Affiliations:** 1 Musculoskeletal Physiotherapy, Ravi Nair Physiotherapy College, Datta Meghe Institute of Medical Sciences, Wardha, IND

**Keywords:** pain, coupling medium, ayurvedic herbs, exercise therapy, phonophoresis, osteoarthritis

## Abstract

Osteoarthritis is a disabling condition globally, affecting a major population. The non-invasive conservative management of osteoarthritis is majorly catered to by physical therapy rehabilitation. Research has been conducted to evaluate the impact of the most commonly used electrotherapy modality, therapeutic ultrasound, on reducing pain and increasing functional activities in individuals suffering from osteoarthritis, but the condition is still, after over two decades of research, growing rapidly in its prevalence. Therefore, the aim of our study was to analyze the literature and compare the evolving trends in coupling medium used for the application of therapeutic ultrasound in arthritic conditions of musculoskeletal origin. Databases of PubMed, Web of Science, Embase, Pedro, and Cochrane were searched till June 2022. The outcome measures used were to detect the status of pain and improvement in functional status. Overall ultrasound therapy adjunct to exercise program was found to be superior to either ultrasound therapy or exercise program alone for the management of pain and functional status of the patients. Additionally, phonophoresis was deduced to have shown better pain relief than conventional ultrasound. Phonophoresis was done using non-steroidal anti-inflammatory drugs and Ayurvedic medicinal herbs in the form of Nano gel.

## Introduction and background

Globally, a leading cause of functional disability due to knee pain is osteoarthritis (OA) of the knee [1]. In India, the prevalence of this ailment ranges from 22% to 39%, with females being more affected than men with increasing age [2]. This significant public health problem burdens the community as it not only deteriorates the physical aspect of individuals' life but also impacts psychosocial health. The resources of the community and that of the affected family are also subjected to the burden of the disease [3]. Therefore, a lot of research has been done for effective prevention, treatment, and rehabilitation in terms of pharmacological and non-pharmacological management of the disease.

Osteoarthritis of the knee is characterized by degenerative wearing and tearing of the articulating surfaces of the tibia-femoral joint. It involves the degradation of cartilage followed by a reduction of joint space more medially in the joint than laterally. As the condition progress, there is the formation of osteophytes and inflammation of the synovial fluid. All these pathological changes are reflected as pain, stiffness, reduced motion in the knee complex, and eventually increased dependency [4].

In the last decade, non-pharmacological physiotherapeutic management of knee osteoarthritis in the early stages gained enough evidence to make it an adjunct along the first line of treatment for the disease [5,6]. Resistance exercise programs improve muscle strength and re-balance of leg muscle activation patterns. These open and closed chain exercises further enhance articular surface loading, reducing knee OA symptoms [7,8]. Aerobic exercises of 30 minutes, including anti-gravity treadmill training, have also improved spatial-temporal parameters, knee flexion/extension gait pattern, and corresponding muscle strength, thereby restoring certain community activities [9].

Ultrasound, an electrotherapeutic modality with low frequency, is found to reduce pain and improve the functioning of the joint in mild to severe cases of knee pain and has been the first choice for conservative non-invasive management [10]. Phonophoresis, an advanced form of ultrasound therapy that involves topical application of the drug to increase its percutaneous absorption, has been used widely as an alternative to oral non-steroidal anti-inflammatory as per the recommendation of the Osteoarthritis Research Society International [11]. A wide range of drugs, including piroxicam, diclofenac, and dexamethasone [12], have been used in studies to evaluate the efficacy of phonophoresis. There have been trials that have used *Phylanthas amarus*, a Chinese medical herb for phonophoresis [13].

Our study aims to combine the results of previous clinical and control trials and analyze the efficacy and safety of different exercise therapies and phonophoresis adjunct with exercises and to further co-relate the effectiveness of phonophoresis with pharmacological agents as well as with non-pharmacological agents.

## Review

Search strategy

Preferred Reporting Items for Systematic Reviews and Meta-Analyses (PRISMA) guidelines were followed for implementing the systematic review and analysis. Databases of PubMed, Web of Sciences, Cochrane, and Embase were searched systematically for articles till June 2022 using appropriate keywords like "knee osteoarthritis", "phonophoresis", "randomized control trial", "exercise therapy", and "Ayurvedic herbs". We extracted additional information from the references of the referred articles.

Diagnosis and clinical features

Diagnosis of osteoarthritis is based on physical examination, medical history, and X-ray [2]. Kallgren and Laurence's scale is the most widely used scale to determine the severity of osteoarthritis radiographically [14]. Even though the scale is widely used, there is a poor correlation between pain severity and osteoarthritis symptoms with the changes seen on the radiograph [15]. Osteoarthritis of the knee is gradual in sudden, beginning after the latter half of the third decade of life, with pain as a primary symptom of concern [3]. Progression of the condition leads to difficulty in walking, cross-leg sitting, and ascending/descending stairs. These symptoms in the elderly above the age of 65 are associated with anxiety, lack of sleep due to pain, and some dependency on others for activities of daily living (ADL) [16]. In a few individuals, associated comorbidities can further downgrade the level of physical activity [17]. Research has shown that the level of physical activity is comparatively low compared to a healthy individual, and it fails to meet the daily recommended levels of activity as they try to avoid activities that cause intermittent bouts of high-intensity pain [18].

Etiology and pathogenesis

Osteoarthritis leads to progressive loss of the synovial properties of the joint, which it affects due to a broad spectrum of interlinked pathological pathways. Articular cartilage and subchondral bone are the most affected structures [19]. An altered bone loading mechanism, either due to sustained injuries or biomechanical abnormalities, leads to proteolytic destruction of the cartilage matrix and chondrocyte death. An imbalance of various chemical substances like cytokines, prostaglandins, and growth factors also accelerates these processes [20]. This leads to abnormal synovial fluid viscosity and, therefore, a phasic inflammation in and around the joint begins. Further activation of nociceptors leads to clinical features of osteoarthritis such as pain, reduced exercise, impaired proprioception, and joint laxity. All these changes add up to the already altered joint biomechanics.

Physical activity and osteoarthritis

Physical activities of different intensities, types, and duration have been found to have inconsistent effects on joints. Weight-bearing activities, especially those that stress the knee joint, have been found to positively impact the articular cartilage of the joint in most cases and, therefore, reduce the risk of developing osteoarthritis in the early stages of life [21].

Articular cartilage undergoes deformation during different activities. High-impact activities like running and squatting have been shown to decrease the thickness of articular cartilage for a short period. Studies have shown that long-term physical activities initiate a chondrogenic response in the cartilage. Studies have shown that patients with chronic illnesses like spinal cord injury and ankle fractures that lead to long-term non-weight-bearing status have a reduced cartilage thickness post-six months compared to pre-injury or initial stages post-injury [22]. It has also been seen that young athletes and endurance runners have a higher thickness of articular cartilage than those compared with same-age-matched adults. The glycosaminoglycan content of the cartilage has also been found to be greater in active individuals with a suitable cartilage thickness and therefore withstand higher mechanical loads [23].

Different studies have concluded to have a negative effect or an increased risk for the development of osteoarthritis in individuals who are elite athletes. It has been hypothesized that it is not the prolonged duration/decade of physical activity that an individual undergoes but the increased chances or number of knee injuries that cause osteoarthritis in such athletes. Following an injury, the weight-bearing mechanism is altered, which has been attributed as the primary cause. ADL have also been studied to affect the knee. It has been seen that be it specific activities or cumulative ADL, a very moderate risk of developing osteoarthritis is present with those activity levels [24].

Physiotherapeutic rehabilitation: exercises

Aerobic exercises have been found to have a more positive effect than that strengthening exercises in reducing pain in a multifactorial way [25]. Resistance training is done with the help of free weights, Thera band (Performance Health, Akron, Ohio, United States), and pulleys, which all improve muscle strength. Low-intensity closed kinetic chain exercises for improving lower limb strength for eight weeks have been found to affect pain, range of motion, and balance positively. Muscle activation exercises for hamstrings, quadriceps, gastrocnemius-soleus, glutes, and transverse abdominus play a significant role in the initial phase of rehabilitation [26].

Physiotherapy rehabilitation: electrotherapy modalities

Electrotherapeutic modalities are widely used as adjuncts to exercise therapy. The various modalities are: Interferential therapy (IFT) and Ultrasound.

IFT utilizes two medium frequency low amplitude currents to travel through the skin and form a beat frequency current with a relatively high amplitude. It has been shown to reduce pain for a short period [27]. In several randomized control trials, transient electrical nerve stimulation (TENS) has been shown to be effective but only when given alongside exercises. Also, the data is insufficient to mark its superiority over other treatment modalities [28].

Ultrasound, which uses sound waves to generate heat within the tissues, is a modality of choice for treating knee osteoarthritis. The thermal and non-thermal effects are beneficial when ultrasound is applied at low intensity, i.e. less than 100 mW/cm2 at a frequency of 1.5 MHz. A frequency of 1-3 MHz is considered beneficial for healing and reducing inflammation as it reaches a depth of 5-3 cms, respectively. A frequency higher or lower than this in research is either non-benefiting or harmful to the tissues. Along with all the above parameters, the aqua sonic gel is used as a conducting medium for transferring waves through the skin [29]. Ultrasound in more recent studies has been advocated with a topically used pharmacological agent like diclofenac (a non-steroidal anti-inflammatory drug (NSAID)) [30]. The transdermal delivery of such drugs through ultrasound has led to a more beneficial effect in improving quality of life [31].

More recent studies have shown the use of Chinese herbal medicines, *Phyllanthus Amarus* nano-particle gel, instead of diclofenac. As these herbal medicines are more widely accepted with almost negligible adverse effects, they are a more widely, readily accepted mode of treatment [13]. In the absence of this gel, transferring waves produced through mechanical vibrations to have a thermal and non-thermal effect is impossible. Therefore, the aim has been on evolving the various conducting mediums to provide the most effective way of ultrasound application where attenuation of the waves is the least and has an added benefit for rehabilitation like facilitation of wound healing and pain reduction.

Phonophoresis and various medicinal drugs

The skin has been used as a route for the administration of pharmacological agents for a long time. Many drug delivery techniques that use energy to enhance absorption and penetration of these agents through the skin have been explored. Clinical evidence shows that ultrasound helps increase the permeability/penetration of pharmacologically active drugs [31]. This process is known as phonophoresis. The microstreaming and cavitation effect of ultrasound leads to an increase in the permeability of the stratum corneum. Over a while, different conducting mediums have been used for this purpose (Table 1). Aqua sonic gel, the most commonly used, has no other healing properties and acts solely as a transfer medium. Diclofenac sodium phonophoresis and thiocolchicoside are muscle relaxants used as topical agents in phonophoresis. It decreases pain and improves functional status in knee osteoarthritis [32]. Similarly, piroxicam gel was shown to be safe and efficacious for treating musculoskeletal pain and is available in Thailand and affordable [11]. But studies have found the application of these drugs has gastrointestinal side effects (minimal). This led to further research utilizing nonpharmacological natural agents to be used in the form of nanogels having anti-inflammatory and analgesic properties in ultrasound phonophoresis.

**Table 1:** Attenuation coefficients of coupling mediums used in ultrasound The attenuation coefficient is one of the parameters used to measure transmissivity of a medium. It represents how easily a material can be penetrated or gives the quantification of how much a beam is weakened by the material through which it is passing [36]. The Aqua sonic gel was found to have the highest transmission [37].

Coupling Medium	Attenuation Coefficient (Np/m)
White petroleum	2.70
Mineral oil	2.15
Water	1.65
Aqua sonic gel	1.62

*P. amarus*, a herb known as "Bhuiavla" in Hindi, is an ayurvedic medicinal plant with flavonoids, tannins, and alkaloids. Over the decade, its oral intake and topical application have been highly valued for its anti-inflammatory, analgesic, antihypertensive, antidiabetic, and antimicrobial properties [33]. It also has effectiveness in protecting against nephrotoxicity induced by other drugs. Pinkaew et al. evaluated the *P. amarus* nanogel phonophoresis effect compared to conventional ultrasound on perceived pain and a six-minute walk test (6MWT). This gel was prepared by mixing the *P. amarus* with standard gel in a ratio of 4/11 by volume [33]. The study concluded a significant difference in pain with a significant walking distance, suggesting that nanoparticle gel is a possible treatment approach worth a better outcome. This has led researchers to utilize ginger, ashwagandha, turmeric, and other medicinal plants as a part of treatment [34,35].

Limitations

Only a small number of the available literature related to phonophoresis was included in this review. The studies that were reviewed evaluated the efficacy of the phonophoresis technique and compared two different agents used for phonophoresis. However, articles were also included that provided information on osteoarthritis for a brief understanding of what the condition is and why phonophoresis is an important aspect of rehabilitation. As a result of the inclusion of the brief introduction related to the condition, this article has a relatively small discussion that focuses mainly on the benefits and properties of coupling gels and their adjuncts.

## Conclusions

Physical activity has a positive effect on joint cartilage. It may, to some extent, reduce the chances of the development of osteoarthritis. Maximum benefit is attained when this regular physical activity is an adjunct to electrotherapeutic treatment for the initial phase of rehabilitation. Ultrasound or phonophoresis is the widely used modality for treating the disabling symptom pain, which is initially intermittent pain and later becomes the continuous dull aching background pain in the knee. Based on this review, we suggest that using plants with medicinal properties along with coupling gels will have an added benefit to phonophoresis. 

The primary benefit of these plant-based medicines is that they have few side effects and added therapeutic properties. But longer duration studies such as cohort and case-control studies are required to obtain adequate knowledge about therapeutic quantities of these bio-medicinal mixtures for rehabilitation purposes. More clinical evidence is also required to evaluate the delay of progression of such condition if a standard set level of physical activity is performed daily, considering all the other confounding variables.

## References

[1] Bowden JL, Hunter DJ, Deveza LA (2020). Core and adjunctive interventions for osteoarthritis: efficacy and models for implementation. Nat Rev Rheumatol.

[2] Pal CP, Singh P, Chaturvedi S, Pruthi KK, Vij A (2016). Epidemiology of knee osteoarthritis in India and related factors. Indian J Orthop.

[3] Singh A, Das S, Chopra A (2022). Burden of osteoarthritis in India and its states, 1990-2019: findings from the Global Burden of disease study 2019. Osteoarthritis Cartilage.

[4] Conaghan PG, Cook AD, Hamilton JA, Tak PP (2019). Therapeutic options for targeting inflammatory osteoarthritis pain. Nat Rev Rheumatol.

[5] Jamtvedt G, Dahm KT, Christie A, Moe RH, Haavardsholm E, Holm I, Hagen KB (2008). Physical therapy interventions for patients with osteoarthritis of the knee: an overview of systematic reviews. Phys Ther.

[6] Davis AM (2012). Osteoarthritis year in review: rehabilitation and outcomes. Osteoarthritis Cartilage.

[7] Sale DG (1988). Neural adaptation to resistance training. Med Sci Sports Exerc.

[8] Vincent KR, Vincent HK (2012). Resistance exercise for knee osteoarthritis. PM R.

[9] Liang J, Lang S, Zheng Y (2019). The effect of anti-gravity treadmill training for knee osteoarthritis rehabilitation on joint pain, gait, and EMG: case report. Medicine (Baltimore).

[10] Draper DO, Klyve D, Ortiz R, Best TM (2018). Effect of low-intensity long-duration ultrasound on the symptomatic relief of knee osteoarthritis: a randomized, placebo-controlled double-blind study. J Orthop Surg Res.

[11] Luksurapan W, Boonhong J (2013). Effects of phonophoresis of piroxicam and ultrasound on symptomatic knee osteoarthritis. Arch Phys Med Rehabil.

[12] Toopchizadeh V, Javadi R, Eftekhar Sadat B (2014). Therapeutic efficacy of dexamethasone phonophoresis on symptomatic knee osteoarthritis in elderly women. IJWHR.

[13] Pinkaew D, Kiattisin K, Wonglangka K, Awoot P (2020). Phonophoresis of Phyllanthus amarus nanoparticle gel improves functional capacity in individuals with knee osteoarthritis: a randomized controlled trial. J Bodyw Mov Ther.

[14] Odole AC, Ekediegwu EC, Issa MU (2019). Differences in clinical variables and physical activity levels of patients with knee osteoarthritis in Ibadan, Nigeria. J Musculoskelet Disord Treat.

[15] Kloppenburg M, Berenbaum F (2020). Osteoarthritis year in review 2019: epidemiology and therapy. Osteoarthritis Cartilage.

[16] Vos T, Flaxman AD, Naghavi M (2012). Years lived with disability (YLDs) for 1160 sequelae of 289 diseases and injuries 1990-2010: a systematic analysis for the Global Burden of Disease Study 2010. Lancet.

[17] Shim HY, Park M, Kim HJ, Kyung HS, Shin JY (2018). Physical activity status by pain severity in patients with knee osteoarthritis: a nationwide study in Korea. BMC Musculoskelet Disord.

[18] Bennell K, Hinman RS, Wrigley TV, Creaby MW, Hodges P (2011). Exercise and osteoarthritis: cause and effects. Compr Physiol.

[19] Couchourel D, Aubry I, Delalandre A, Lavigne M, Martel-Pelletier J, Pelletier JP, Lajeunesse D (2009). Altered mineralization of human osteoarthritic osteoblasts is attributable to abnormal type I collagen production. Arthritis Rheum.

[20] Man GS, Mologhianu G (2014). Osteoarthritis pathogenesis - a complex process that involves the entire joint. J Med Life.

[21] Tse AC, Wong TW, Lee PH (2015). Effect of low-intensity exercise on physical and cognitive health in older adults: a systematic review. Sports Med Open.

[22] Smith JK (2020). Exercise as an adjuvant to cartilage regeneration therapy. Int J Mol Sci.

[23] Reginster JY, Neuprez A, Lecart MP, Sarlet N, Bruyere O (2012). Role of glucosamine in the treatment for osteoarthritis. Rheumatol Int.

[24] Nobi MG, Azad AK, Ahmed B, Rashid I, Islam T, Shakoor M (2012). Effects of activities of daily living (ADL) instructions on patient with osteoarthritis of the knee. J Med.

[25] Zhang W, Moskowitz RW, Nuki G (2008). OARSI recommendations for the management of hip and knee osteoarthritis, part II: OARSI evidence-based, expert consensus guidelines. Osteoarthritis Cartilage.

[26] Abdelraouf OR, Abdel-Aziem AA, Ahmed AA, Nassif NS, Matar AG (2019). Backward walking alters vastus medialis oblique/vastus lateralis muscle activity ratio in females with patellofemoral pain syndrome. Turk J Phys Med Rehabil.

[27] Eftekharsadat B, Babaei-Ghazani A, Habibzadeh A, Kolahi B (2015). Efficacy of action potential simulation and interferential therapy in the rehabilitation of patients with knee osteoarthritis. Ther Adv Musculoskelet Dis.

[28] Mugheeb TM, Al-Shehri A, Amoudi KOA (2021). Effect of TENS in management of knee osteoarthritis-a systematic review. Int J Recent Innov Med Clin.

[29] Yadollahpour A, Jalilifar M, Rashidi S, Rezaee Z (2014). Ultrasound therapy for wound healing: a review of current techniques and mechanisms of action. J Pure Appl Microbiol.

[30] Zhou XY, Zhang XX, Yu GY (2018). Effects of low-intensity pulsed ultrasound on knee osteoarthritis: a meta-analysis of randomized clinical trials. Biomed Res Int.

[31] Azagury A, Khoury L, Enden G, Kost J (2014). Ultrasound mediated transdermal drug delivery. Adv Drug Deliv Rev.

[32] Fernández-Cuadros ME, Casique Bocanegra L, Florin MJ, Rabasa S, Pérez Moro O (2020). Effect of diclofenac gel phonophoresis on temporomandibular joint disorders: a prospective quasi-experimental study. Middle East J Rehabil Health Stud.

[33] (2022). Phyllanthus amarus. https://www.sciencedirect.com/topics/agricultural-and-biological-sciences/phyllanthus-amarus.

[34] Balkrishna A, Sakat SS, Joshi K (2019). Anti-inflammatory and anti-arthritic efficacies of an indian traditional herbo-mineral medicine "divya amvatari ras" in collagen antibody-induced arthritis (CAIA) mouse model through modulation of IL-6/IL-1β/TNF-α/NFκB signaling. Front Pharmacol.

[35] Ghildiyal S, Gautam MK, Joshi VK, Goel RK (2013). Anti-inflammatory activity of two classical formulations of Laghupanchamula in rats. J Ayurveda Integr Med.

[36] Kore PS, Pawar PP (2014). Measurements of mass attenuation coefficient, effective atomic number and electron density of some amino acids. Radiat Phys Chem.

[37] Casarotto RA, Adamowski JC, Fallopa F, Bacanelli F (2004). Coupling agents in therapeutic ultrasound: acoustic and thermal behavior 1,2. Arch Phys Med Rehabil.

